# Supplementary material for: Uncertainty Evaluation of Soil Heavy Metal(loid) Pollution and Health Risk in Hunan Province: A Geographic Detector with Monte Carlo Simulation
Source: Toxics. 2023 Dec 8;11(12):1006. doi: 10.3390/toxics11121006 (PMC10747857; doi:10.3390/toxics11121006)
Supplement: Supplementary file 1 [file toxics-11-01006-s001.zip › toxics-2745220-supplementary.pdf]

toxics

MDPI

Supplementary Materials: Uncertainty Evaluation of Soil Heavy Metal(loid) Pollution and Health Risk in Hunan Province: A Geographic Detector with Monte Carlo Simulation

Baoyi Zhang, Yingcai Su, Syed Yasir Ali Shah and Lifang Wang

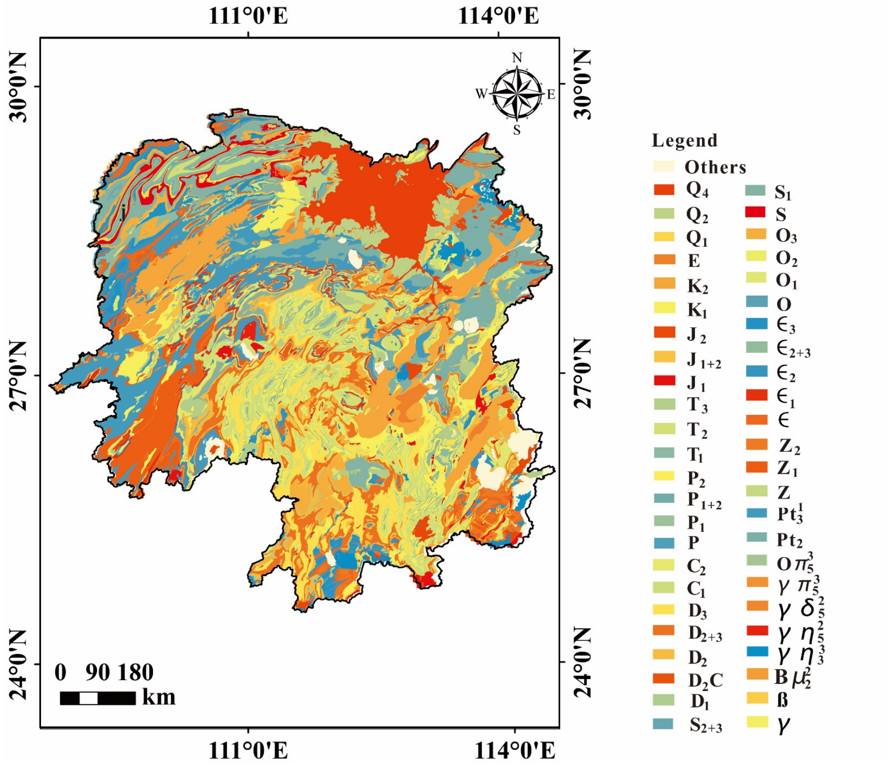

Figure S1. Geological map of Hunan Province. Lithological units in legend are presented in Table S1.

Table S1. Lithostratigraphic types.

Lithological unit	Description	Geological era
Q_4	Quaternary Holocene alluvium and lacustrine deposits	Quaternary
Q_2	Quaternary Holocene alluvium and lacustrine deposits	Quaternary
Q_1	Lake deposit I: feldspathic coarse sand, clay, peat. Alluvial f: gravel	Quaternary
E	Union layer	Paleogene
K_2	Mesozoic Upper Cretaceous red sandstone, calcareous mudstone, and sandy conglomerate	Cretaceous
K_1	Red siltstone, mudstone, sand conglomerate, basalt	Cretaceous
J_2	Red mudstone, silty conglomerate, sandy limestone, conglomerate	Jurassic
J_{1+2}	Sandstone, feldspar quartz sandstone, red mudstone, siltstone	Jurassic
J_1	Sandstone, feldspar quartz sandstone, shale, conglomerate, basalt	Jurassic
T_3	Conglomerate, mudstone, shale, quartz conglomerate	Triassic
T_2	Variegated calcareous mudstone, siltstone, dolomite	Triassic

T_1	Sandstone, mudstone, shale, quartz conglomerate, coal bearing	Triassic
P_2	Longtan Formation and Wujiaping limestone union	Permian
P_{1+2}	Liangshan Formation, Qixia limestone, Maokou limestone, Longtan Formation, Dalong Formation	Permian
P_1	Liangshan Formation, Qixia Formation, Maokou limestone union	Permian
P	Upper Series: siliceous rock, shale, limestone, sandstone<br>Lower Series: Chert limestone, magnesium marl	Permian
C_2	Limestone, marl, sandstone, shale	Carboniferous
C_1	Limestone, dolomite	Carboniferous
D_2C	Middle and upper Devonian and Carboniferous co-stratification	-
D_3	Marl, limestone, sandstone, shale, hematite	Devonian
D_{2+3}	Union layer	Devonian
D_2	The upper part is limestone and dolomite; The lower part is sandstone and sand conglomerate	Devonian
D_1	Purple sandstone, shale, sand conglomerate	Devonian
S_{2+3}	Union layer	Siluric
S_1	Shallow metamorphic fine sandstone, slate	Siluric
S	Siltstone, shale, sandy limestone, shell limestone	Siluric
O_3	Black shale, siliceous rock, feldspar quartz sandstone, shale	Ordovician
O_2	Limestone, marl, shallow metamorphic siltstone	Ordovician
O_1	Limestone, dolomite with shale, calcareous slate, shallow metamorphic siltstone	Ordovician
O	Union layer	Ordovician
Є$_3$	Dolomite, lime dolomite, limestone	Cambrian
Є$_{2+3}$	Middle series and upper series parallel layer	Cambrian
Є$_2$	Limestone, dolomite and shale	Cambrian
Є$_1$	Limestone, marl, shale, carbonaceous shale	Cambrian
Є	Union layer	Cambrian
Z_2	Limestone, dolomite, siliceous rock, mafic rock	Sinian
Z_1	Pebbly mudstone, slate, arkose	Sinian
Z	Union layer	Sinian
Pt_3^1	Nishi Group: metamorphic sandstone, gravel sandstone, slate<br>Banxi Group: purple red slate, metamorphic sandstone, limestone, sand-bearing conglomerate<br>Gaojian Group: black slate, metamorphic sandstone, tuff, sand containing conglomerate	Upper Proterozoic
Pt_2	Lengjiaxi Group: slate, metamorphic sandstone, andesite, basalt, volcanic rock	Mesoproterozoic
$O\pi_5^3$	Quartz porphyry	Yanshanian Cretaceous
$\gamma\pi_5^3$	Granite porphyry	Yanshanian Cretaceous
$\gamma\eta_5^2$	Monzonitic granite	Yanshanian Jurassic
$\gamma\delta_5^2$	Granodiorite	Yanshanian Jurassic
$\gamma\eta_3^3$	Granodiorite	Caledonian Silurian System
$B\mu_2^2$	Diabase	Middle Proterozoic
β	Basalt	-
γ	Granite	-

Table S2. Statistics characteristics of the heavy metal(loid)s ($mg \cdot kg^{-1}$).

	Cr	Cd	As	Pb	Hg
Mean±SD	75.65±30.45	0.77±4.18	19.18±43.94	57.96±144.08	0.18±1.61
Median	71.96	0.41	14	39.9	0.12
Minimum	2.71	0	0.67	1.51	0
Maximum	1281	417.5	3782.1	9763	235.7
Skewness	7.2	5.3×10^1	3.4×10^1	3.4×10^1	1.1×10^2
Kurtosis	2.1×10^2	1.8×10^3	3.8×10^3	1.9×10^3	1.3×10^4
C.V.	0.4	5.41	2.29	2.49	8.94
Background values	67 [1]	0.08 [1]	14 [1]	27 [1]	0.09 [1]
Risk control standard	150	0.3	20	70	0.5

Table S3. Risk screening values for soil contamination of agricultural land (($mg \cdot kg^{-1}$).

Pollutant	Type	pH≤5.5	5.5<pH≤ 6.5	6.5<pH≤7.5	pH>7.5
Cr	Paddy	250	250	300	350
	Others	150	150	200	250
Cd	Paddy	0.3	0.4	0.6	0.8
	Others	0.3	0.3	0.3	0.6
As	Paddy	30	30	25	20
	Others	40	40	30	25
Pb	Paddy	80	100	140	240
	Others	70	90	120	170
Hg	Paddy	0.5	0.5	0.6	1.0
	Others	1.3	1.8	2.4	3.4

Table S4. The classification criteria for the assessment of soil heavy metal(loid) pollution assessment.

Level	Nemerow index	Degree of Contamination
1	P_n<=0.7	Uncontaminated
2	0.7<P_n<=1.0	Warning Level of Caution
3	1.0<P_n<=2.0	Low contamination
4	2.0<P_n<=3.0	Moderate contamination
5	P_n>3.0	High contamination

Table S5. The probabilistic distribution of exposure parameters for human health risk assessment.

Parameters	Unit	Probabilistic Distribution	Children	Adults	Reference
Ingestion rate (IR_{ing})	$mg \cdot d^{-1}$	lognormal	50th: 113.6<br>95th: 275.5	50th: 72.2<br>95th: 187.6	[2]
Exposure frequency (EF)	$d \cdot year^{-1}$	point	350	350	[3]
Exposure duration (ED)	year	uniform	(5,6)	(19-44)	[2]
Skin adherence factor (AF)	$mg \cdot cm^{-1}$	Beta	0.2(0, 3.3)	0.07(0, 0.3)	[4]
Skin area exposed to soils (SA)	cm^2	Lognormal	50th: 7970.8<br>95th: 9290.1	50th: 15922.0<br>95th: 18730.0	[2]
Dermal adsorption factor (ABS)	–	point	0.001(As:0.03)	0.001(As:0.03)	[4]
Average body weight (BW)	kg	lognormal	50th:19.3<br>95th:24.5	50th:60.1<br>95th:80.6	[2]
Average time (AT)	NCR(d)<br>CR(d)	point<br>point	ED×365<br>70×365	ED×365<br>70×365	[4]

Table S6. References dose (RfD, mg·(kg·d)$^{-1}$) and slope factor (SF, mg·(kg·d)$^{-1}$) of human health risk assessment through different pathways.

Parameters	As	Cd	Cr	Pb	Hg
RfD_{ing}	3.00×10^{-4}	1.00×10^{-3}	3.00×10^{-3}	3.50×10^{-3}	3.00×10^{-4}
RfD_{dermal}	1.23×10^{-4}	1.00×10^{-5}	6.00×10^{-5}	5.25×10^{-4}	2.14×10^{-5}
SF_{ing}	1.50	5.01×10^{-1}	5.00×10^{-1}	–	–
SF_{dermal}	3.66	20	20	–	–
Reference	[5]	[5]	[3]	[5]	[6]

Table S7. The interaction type and criterion between factor X_1 and factor X_2.

	Type of Interaction	Judging Description
1	Nonlinearly weakened	q(X1∩X2) < Min(q(X1), q(X2))
2	Univariate nonlinearly weakened	Min(q(X1), q(X2)) < q(X1∩X2) < Max(q(X1), q(X2))
3	Bivariate enhanced	q(X1∩X2) > Max(q(X1), q(X2))
4	Independent	q(X1∩X2) = q(X1) + q(X2)
5	Nonlinearly enhanced	q(X1∩X2) > q(X1) + q(X2)

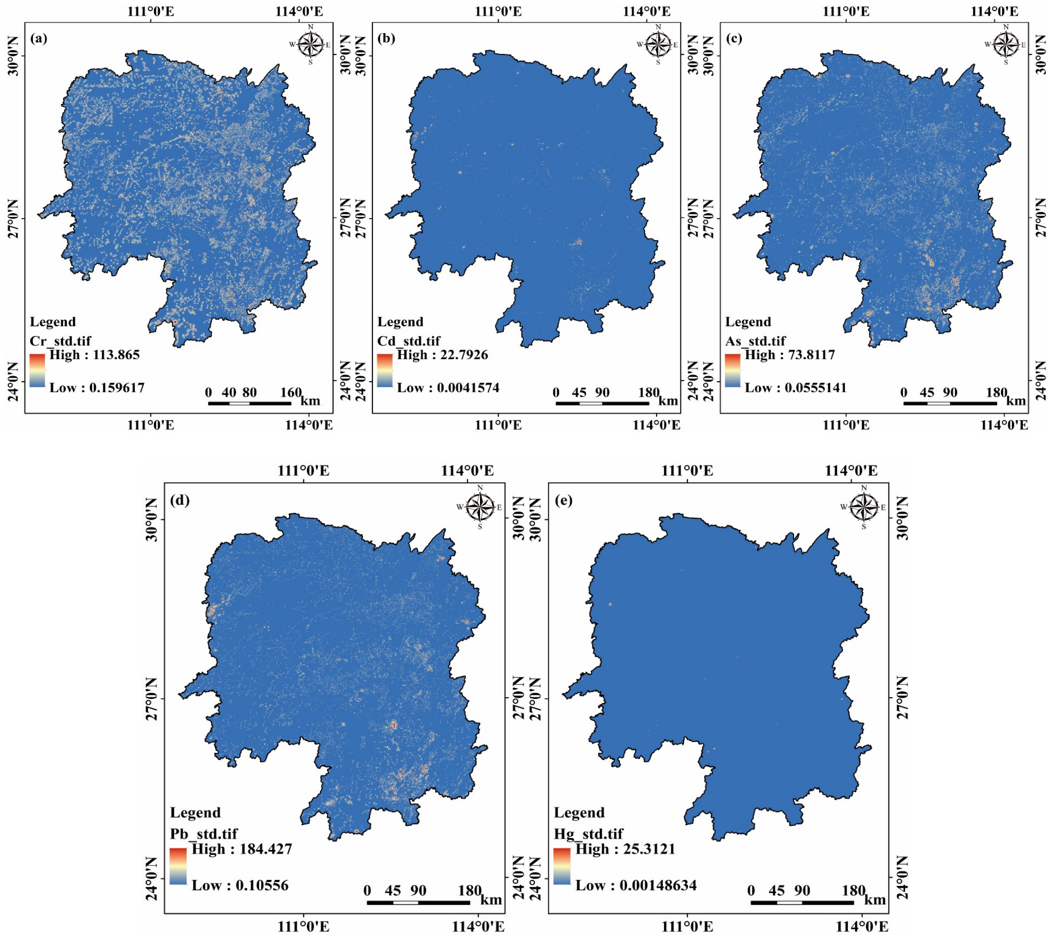

Figure S2. Uncertainty of location and concentration of heavy metal(loid)s of (a) Cr, (b) Cd, (c) As, (d) Pb, and (e) Hg.

References

1. Li, X.Z.; Zhao, Z.Q.; Yuan, Y.; Wang, X.; Li, X.Y. Heavy metal accumulation and its spatial distribution in agricultural soils: Evidence from Hunan province, China. *RSC Adv.* **2018**, *8*, 10665–10672. https://doi.org/10.1039/c7ra12435j.
2. Zhang, H.; Zhang, F.; Song, J.; Tan, M.L.; Kung, H.-t.; Johnson, V.C. Pollutant source, ecological and human health risks assessment of heavy metals in soils from coal mining areas in Xinjiang, China. *Environ. Res.* **2021**, *202*, 111702. https://doi.org/10.1016/j.envres.2021.111702.
3. Liu, Z.; Du, Q.; Guan, Q.; Luo, H.; Shan, Y.; Shao, W. A Monte Carlo simulation-based health risk assessment of heavy metals in soils of an oasis agricultural region in northwest China. *Sci. Total Environ.* **2023**, *857*, 159543. https://doi.org/10.1016/j.scitotenv.2022.159543.
4. USEPA. *Exposure Factors Handbook: 2011 Edition*; EPA/600/R-090/052F; U.S. Environmental Protection Agency, Office of Research and Development, National Center for Environmental Assessment: Washington, DC, USA, 2011.
5. Guo, G.; Li, K.; Lei, M. Accumulation, environmental risk characteristics and associated driving mechanisms of potential toxicity elements in roadside soils across China. *Sci. Total Environ.* **2022**, *835*, 155342. https://doi.org/10.1016/j.scitotenv.2022.155342.
6. Liu, H.; Zhang, Y.; Yang, J.; Wang, H.; Li, Y.; Shi, Y.; Li, D.; Holm, P.E.; Ou, Q.; Hu, W. Quantitative source apportionment, risk assessment and distribution of heavy metals in agricultural soils from southern Shandong Peninsula of China. *Sci. Total Environ.* **2021**, *767*, 144879. https://doi.org/10.1016/j.scitotenv.2020.144879.
